# Radiation-induced small extracellular vesicles as “carriages” promote tumor antigen release and trigger antitumor immunity

**DOI:** 10.7150/thno.43539

**Published:** 2020-03-26

**Authors:** Wanzun Lin, Yanyan Xu, Xiaochuan Chen, Jun Liu, Youliang Weng, Qingyang Zhuang, Feifei Lin, Zongwei Huang, Shihong Wu, Jianming Ding, Long Chen, Xianxin Qiu, Lurong Zhang, Junxin Wu, Duo Lin, Sufang Qiu

**Affiliations:** 1 Department of Radiation Oncology, Fujian Cancer Hospital & Fujian Medical University Cancer Hospital, Fuzhou, China; 2 Department of Obstetrics and Gynecology, Shanghai General Hospital, Shanghai Jiao Tong University School of Medicine, Shanghai, China.; 3 Department of Radiation Oncology, Fujian Medical University Cancer Hospital & Fujian Cancer Hospital, Fuzhou, China; 4 Department of Oncology, Fujian Cancer Hospital & Fujian Medical University Cancer Hospital, Fuzhou, China; 5 Division of Neurocritical Care, Huashan Hospital, Fudan University, Shanghai, China; 6 Department of Radiation Oncology, Shanghai Proton and Heavy Ion Center, Shanghai, China; 7 Department of radiobiology, Fujian Cancer Hospital & Fujian Medical University Cancer Hospital, Fuzhou, China; 8 Key Laboratory of OptoElectronic Science and Technology for Medicine, Ministry of Education, Fujian Provincial Key Laboratory for Photonics Technology, Fujian Normal University, Fuzhou, China; 9 Fujian Provincial Key Laboratory of Translational Cancer Medicine, Fuzhou, China

**Keywords:** small extracellular vesicles, antitumor immunity, radiation therapy, abscopal effect, tumor-associated antigens

## Abstract

**Rationale**: Accumulating evidence supports the importance of radiation therapy in the induction of antitumor immunity. Small extracellular vesicles (sEVs) play essential roles in tumor antigen loading and delivery. However, the role of sEVs in radiation-induced antitumor immunity remains unclear. It is therefore important to determine the role and regulatory mechanisms of sEVs in radiation-induced immunity.

**Methods**: Tumor cells were irradiated (8 Gy), and sEVs were purified via ultracentrifugation. Primary tumor and experimental lung metastasis models were established in mice to evaluate antitumor immunity triggered by immunization with sEVs. Proteomic and bioinformatic analyses were performed to identify altered cargos in sEVs induced by radiation. Peptides derived from up-regulated proteins in sEVs were designed and synthesized as vaccines according to major histocompatibility complex (MHC) I binding and immunogenicity.

**Results**: Here, we demonstrated that sEVs derived from irradiated tumor cells could trigger antitumor immunity against primary tumor and experimental lung metastasis by enhancing CD8^+^ and CD4^+^ T cell infiltration. Radiation may also enrich sEVs with tumor antigens and heat-shock proteins. Furthermore, CUB domain-containing protein 1 (CDCP1) derived from radiation-induced sEVs was identified as a novel tumor-associated antigen and developed as a peptide vaccine that may generate antitumor immune responses.

**Conclusions**: Our results demonstrate that the use of sEVs secreted by irradiated tumor cells constitutes an efficient approach for tumor antigen delivery and presentation and highlight the role of sEVs in radiation-triggered antitumor immunity.

## Introduction

Radiotherapy (RT) is currently an important strategy for cancer treatment. Accumulating evidence supports the role of RT in the induction of antitumor immunity 1. The immune-mediated antitumor effects of RT may trigger the regression of metastatic tumors that are distant from the irradiated field, which is known as the “abscopal effect” 2. However, the underlying mechanisms of radiation-induced activation of the immune system remain to be fully elucidated. One of the critical mechanisms is the potential of radiation therapy to convert the irradiated tumors into *in situ* vaccines. This results in the liberation of tumor cell-derived antigens and damage-associated molecular patterns (DAMPs), and causes modulation of the tumor microenvironment by promoting dendritic cell (DC) recruitment and T cell priming 3.

Small extracellular vesicles (sEVs) are nanometric vesicles (50-200 nm in diameter) formed in vesicular bodies in the endosomal network that can be released by almost all types of cells, including cancer cells 4. sEVs play important roles in cell communication, both locally and systemically, by exchanging their contents, which include a subset of proteins, lipids, and functional genetic material derived from donor cells 5, 6. sEVs have recently become a research hotspot in cancer immunity. Tumor-derived sEVs act as carriers of native tumor-associated antigens (TAAs) that can be efficiently transferred to DCs and induce antigen- specific CD8+ T cell activation via reprocessing 7. DC vaccine pulsed with sEVs, displaying an array of tumor antigens, can elicit a stronger immune response than that with cell lysates *in vitro* and *in vivo* 8. Furthermore, sEVs carry surface peptide-major histocompatibility complexes (p-MHC) and may therefore directly stimulate CD8 and CD4 T cells.

In this study, we investigated the role of sEVs in RT-induced antitumor immunity. Our results demonstrated that radiation enriched sEVs with tumor antigens and DAMPs. Radiation-induced sEVs acted as efficient carriers by promoting the releasing and presentation of tumor antigens. Antitumor active immunity primed by radiation-induced sEVs could represent an effective, long-lasting, and safe strategy to suppress cancer progression. Our results further elucidate the mechanisms of radiation therapy in the activation of antitumor immunity.

## Materials and Methods

### Ethical committee approval

Animal experiments were approved by the Fujian Medical University Institutional Animal Ethical Committee (FJMU IACUC #2018-075). The applicable institutional guidelines for the care and use of animals were followed.

### Cell culture

Murine hepatoma H22 and murine breast cancer 4T1 cells were purchased from Beina Chuanglian Biotechnology Institute (Beijing, China) and were cultured in RPMI-1640 medium (cat# 11835055, Invitrogen, Carlsbad, CA, USA) with 10% sEVs-free fetal bovine serum (FBS; cat# UR50202, Umibio, Shanghai, China) and 1% penicillin-streptomycin (cat# 15140155, Invitrogen).

### sEVs Isolation

sEVs were isolated via ultracentrifugation. Briefly, the culture supernatants of 8 Gy-irradiated and non-irradiated H22 or 4T1 cells were centrifuged for 5 min at 500 × g and cells were eliminated from the samples. The supernatants were transferred to new polycarbonate tubes and centrifuged for 10 min at 2000 × g. The supernatants were again collected and transferred to new polycarbonate tubes before centrifuging for 30 min at 10 000 × *g* to eliminate shed microvesicles (200-1000 nm). The supernatants were then collected and filtered through 0.22-μm membrane filters (Merck Millipore, Burlington, MA, USA) before centrifuging for 2 h at 100 000 × g. Finally, sEVs were resuspended in 1× phosphate-buffered saline (PBS) and stored at -80 ℃ until further use.

### sEVs characterization

To investigate the morphological characteristics of sEVs, their size and number were determined using a Multiple-Laser ZetaView® f-NTA Nanoparticle Tracking Analyzer (Particle Metrix, Meerbusch, Germany). The morphology of the sEVs obtained was also observed directly via transmission electron microscopy (TEM; Tecnai 12, Philips, Amsterdam, The Netherlands). Western blotting was performed to analyze the sEV-specific surface markers CD9, CD81, and Alix.

### Transmission Electron Microscopy

Briefly, sEVs were adsorbed onto Formvar- carbon grids. Next, sEVs were fixed using 2% glutaraldehyde for 5 min and washed three times with distilled H_2_O. After washing, the grids were stained with uranyl acetate solution. sEV images were obtained using a Tecnai 12 instrument (Philips) operated at 80 kV.

### Flow cytometry for nanoparticle analysis

sEVs concentration was measured using nanoparticle flow cytometry (NanoFCM, SNA-D1, UK) and corresponding software NF Profession 1.0. Isolated sEVs samples were appropriately diluted using 1× PBS buffer to measure the concentration. The nanoparticle flow cytometry system was calibrated using 200 nm polystyrene particles and the laser was focused by 15/40 mW 488.

### Immunization with sEVs

Female ICR or BALB/c mice (22-25 g; Slaccas Experimental Animal LLC, Shanghai, China; license SCXK 2012-0002) were randomly divided into three groups: (1) PBS-treated control group (n = 10), (2) mice immunized with sEVs secreted by non-irradiated cells (n = 10), and (3) mice immunized with sEVs secreted by 8 Gy-irradiated cells (n = 10). For the ICR hepatoma model, ICR mice were immunized with sEVs derived from H22 cells. For the BALB/c breast cancer model, BALB/c mice were immunized with sEVs derived from 4T1 cells. For groups 2 and 3, 200 μg of each type of sEVs was dissolved in 0.1 mL PBS and subcutaneously injected into each foot and into eight spots on the back of each mouse 9-11.

### Flow cytometry (FCM) analysis for CD8^+^ and CD4^+^ T lymphocytes

At the end of experiment, some of the mice were sacrificed and the lymph nodes and spleen were harvested. Blood lymphocytes were also isolated via density gradient centrifugation using Ficoll. Lymphocytes were stained with fluorescein isothiocyanate (FITC)-anti-mouse CD3 for 1 h, centrifuged at 350 × *g* for 5 min to wash off free antibody, and stained with peridinin chlorophyll protein complex (PerCP)-anti-mouse CD8 or allophycocyanin (APC)-anti-mouse CD4 for 1 h at 4 °C. Double-stained CD3^+^ CD8^+^ and CD3^+^ CD4^+^ T lymphocytes were analyzed using a BD Accuri™ C6 Flow Cytometer (BD Biosciences, Franklin Lakes, NJ, USA).

### Primary tumor model and tumor growth measurement

H22 hepatoma or 4T1 breast cancer cells (1 × 10^6^) were resuspended in 0.2 mL PBS and subcutaneously injected into the backs of mice 25 days after immunization. The tumors in each mouse in the PBS control group, 0 Gy-sEV-immunized group, and 8 Gy-sEV-immunized group were measured using a digital caliper twice per week. Tumor volume was calculated according to the following formula: long diameter × short diameter^2^/2, and a tumor growth curve was plotted as tumor volume versus time. At the end of the experiment, tumors were harvested, photographed, and weighed.

### Experimental metastasis model and *in vivo* lung imaging

4T1 breast cancer cells (1 × 10^6^) transfected with green fluorescent protein and luciferase complimentary (c)DNA and resuspended in 0.3 mL PBS were injected intravenously into the tail vein of BALB/c mice 25 days after immunization. Two weeks later, the growth of 4T1 experimental lung metastases was measured using an IVIS Lumina III *in vivo* imaging system (PerkinElmer, Waltham, MA, USA). Total photon flux (P/S) was also measured. At the end of the study, the lung metastases in each mouse were counted under a dissecting microscope.

### Survival curve analysis

The number of BALB/c mice bearing experimental lung metastases was recorded every day. The percentage survival in each group was calculated as the number of surviving mice/number of the total mice studied × 100% and plotted against time.

### Immunohistochemical (IHC) analysis

H22 and 4T1 tumors were harvested, fixed in 10% formalin overnight, and prepared as 5-μm-thick paraffin sections. The slides were then stained with hematoxylin & eosin or subjected to IHC analysis using anti-mouse CD8 or CD4, followed by 3,3-diaminobenzidine staining. Photographs were obtained under a microscope (200× magnification).

### Cytotoxicity assay

Spleen lymphocytes were harvested 24 days after immunization and co-cultured with H22 or 4T1 tumor cells (tumor cell:lymphocyte ratio = 1:20) in 24-well plates in triplicate, in serum-free RPMI-1640 medium containing 20 U/mL mouse interleukin (IL)-2 (cat #200-02, PeproTech Rocky Hill, NJ, USA) for 2 days. Cytotoxicity was evaluated according to the amount of lactate dehydrogenase released in the medium using an automatic biochemical analyzer P800 (Roche, Basel, Switzerland).

### Label-free quantitative proteomic analysis of differentially expressed proteins in radiation-induced sEVs

4T1 sEVs were collected 48 h after 8 Gy irradiation. Label-free quantitative proteomic analysis was then performed to identify differentially expressed proteins assisted by Aksomics Bioscience Institute (Shanghai, China). Briefly, sEVs lysed in radioimmunoprecipitation assay (RIPA) buffer were supplemented with protease inhibitor cocktail. A total of 100 µg protein was diluted to a concentration of 1 mg/mL using RIPA buffer and precipitated using excess acetone. Precipitated proteins were then subjected to dissolution, reduction, alkylation, trypsinization, and desalination. After cooling, 2 µg digested peptides were further analyzed using a Q Exactive mass spectrometer (Thermo Fisher Scientific, Waltham, MA, USA) connected to an Easy Nano Liquid Chromatography system (Thermo Fisher Scientific). The run time was 120 min per sample. Analysis was performed using a positive ion scanning mode and the scanned parent ion range was 350-1600 m/z. Data-dependent acquisition (DDA) was performed by collecting 20 fragment patterns (MS2 scan, HCD) after each full scan with the following parameters: MS1 resolution at M/Z 200: 70,000; MS2 resolution at M/Z 200: 17,500; MS1 automatic gain control (AGC) at 3 × 106; MS2 AGC at 1 × 105; maximum ion injection time of 50 ms for MS1 and 45 ms for MS2; normalized collision energy at 28%; isolation window at 2.0 m/z; and time of dynamic exclusion at 40s. For peptide and protein identification, tandem mass spectra were identified using the Universal Protein Resource database (Uniprot_rat_2016_09) using MaxQuant v1.5.6.0 (Max Planck Institute, Munich, Germany). The digestion model was trypsin with a maximum of three trypsin missed-cleavage sites. Oxidation of methionine and acetylation of the protein N terminus were set as variable modifications, and carbamidomethyl as fixed modification with a maximum allowance of three variable modifications per peptide. The false discovery rate at the peptide and protein level was set to 0.01.

### Designing CDCP1 peptides to prime active immunity

The programs IEDB (http://www.iedb.org/) and SYFPEITHI (http://www.syfpeithi.com/) were used to design two CDCP1 peptides based on optimal MHC I binding and immunogenicity 12, 13. The amino acid sequences of the two peptides were N′-SYTPYFKEE-C′ and N′-FKEEGIFTVTP-C′. These were synthesized with N-terminal acetylation and C-terminal amidation to prevent peptidase degradation (Top-peptide Biotech LLC, Shanghai, China). Briefly, 100 μg of each peptide dissolved in 0.1 mL PBS was emulsified with an equal volume of Freund's complete adjuvant and was subcutaneously injected into BALB/c mice four times, at 5-day intervals. PBS with an equal volume of Freund's adjuvant was used in the control group.

### FCM analysis for peptide-stimulated active CD8^+^-interferon (IFN)γ^+^ T lymphocytes

Splenic lymphocytes from CDCP1 peptide-immunized and control mice were obtained via density gradient centrifugation using Ficoll. Lymphocytes were stimulated with CDCP1 peptides (10 μg/mL), blocked with 10 μg/mL brefeldin A (cat #S1536, Beyotime, Jiangsu, China) for 2 h, and stained with APC-anti-mouse CD8 for 1 h. The cells were then fixed with 4% paraformaldehyde for 15 min, permeabilized with 0.1% Triton-PBS, and stained with phycoerythrin (PE)-anti-mouse IFNγ for 1 h at 4 ºC. The double-stained CD8^+^-IFNγ^+^ T lymphocytes were analyzed using a BD Accuri™ C6 Flow Cytometer (BD Biosciences).

### Western blot analysis

Briefly, 40 µg protein per sample was analyzed on a 12% gel via sodium dodecyl sulfate-polyacrylamide gel electrophoresis (SDS-PAGE) and transferred to a nitrocellulose membrane. Next, the bands were blocked with 5% bovine serum albumin (BSA), incubated with anti-mouse CD9 (1:1000), CD81 (1:1000), Alix (1:1000), or CDCP1 (1:1000) at 4 °C for 12 h, washed thrice for 15 min each time in Tris-buffered saline with Tween (TBST) at 25 °C, incubated with horseradish peroxidase-conjugated secondary antibody for 2 h at 25 °C, and washed thrice for 15 min each time with TBST. The bands were exposed using a ChemiDoc^TM^ imaging system (Bio-Rad, US) according to the manufacturer's instructions and were further analyzed using the software ImageJ to obtain densitometry values.

### DC culture and identification

Bone marrow cells were obtained from mice femurs by flushing with RPMI 1640 culture medium. After lysing red blood cells, cells were maintained at a density of 2 × 10^5^ cells/mL in complete RPMI 1640 supplemented with 10% sEV-depleted FBS, 1% penicillin/streptomycin, 40 ng/mL granulocyte-macrophage colony-stimulating factor (GM-CSF; R&D Systems, Minneapolis, MN, USA), and 20 ng/mL recombinant mouse IL-4 protein (R&D Systems). Non-adherent cells were depleted after 48 h. The remaining cells were cultured, and the medium was changed every other day. On day 7, the culture comprised mostly immature DCs and these were used for the study. At the end of experiment, the cells were stained with FITC-anti-mouse CD11c for 1 h, centrifuged at 350 × *g* for 5 min to wash off free antibody, and subjected to FCM analysis using a BD Accuri™ C6 Flow Cytometer (BD Biosciences).

### sEV labeling and uptake by DCs

sEVs were labeled with PKH67 (Sigma-Aldrich, St Louis, MO, USA) according to the manufacturer's instructions. Briefly, sEVs were diluted to 0.5 mL using Diluent C from the PKH67 kit. In parallel, 4 μL PKH67 dye was added to 0.5 mL Diluent C and incubated with the sEVs at room temperature for 5 min. Next, 2 mL 10% BSA/PBS was added to stop the reaction. Excess dye was removed via ultracentrifugation at 100,000 × *g* for 2 h, and the labeled sEVs were suspended in PBS and used for uptake experiments. After 24 h incubation with labeled sEVs (10 µg/1 × 10^6^ DCs), DCs were stained with 4′,6-diamidino-2-phenylindole, washed with PBS, and visualized using confocal microscopy.

### Flow Cytometry Cell Sorting

Sorting was performed using a BD FACSAria III flow cytometer (BD Biosciences). Spleen lymphocytes were harvested via density gradient centrifugation using Ficoll. Lymphocytes were stained with FITC-anti-mouse CD8 for 1 h and centrifuged at 350 × *g* for 5 min to wash off free antibody. Fluorescence-activated cell sorting (FACS) was performed at all stages to separate FITC^+^ and FITC^-^-gated populations following exclusion of dead cells.

### DC and CD8^+^ T cell co-culture

A cytotoxic T lymphocytes (CTLs) culture was initiated by incubating CD8^+^ T cells with 0 Gy-sEV- or 8 Gy-sEV-treated DCs at a ratio of 10:1 in complete medium supplemented with 20 U/mL IL-2.

### Antibodies

The following antibodies were used in this study: CD81 rabbit monoclonal Ab (mAb; cat #10037, Cell Signaling Technology, Danvers, MA, USA), Alix rabbit mAb (cat #92880, Cell Signaling Technology), Akt rabbit mAb (cat #4691, Cell Signaling Technology), Phospho-Akt rabbit mAb (cat #4060, Cell Signaling Technology), PI3 Kinase rabbit mAb (cat #4257, Cell Signaling Technology), Phospho-PI3 Kinase rabbit mAb (cat #17366, Cell Signaling Technology), β-Actin rabbit mAb (cat #4970, Cell Signaling Technology), CD9 rabbit mAb (cat #ab92726, Abcam, Cambridge, UK), CD4 rabbit mAb (cat #ab183685, Abcam), CD8 rabbit mAb (cat #ab22378, abcam), CDCP1 rabbit pAb (cat #12754-1-AP, Proteintech, Rosemont, IL, USA), PerCP-anti-mouse CD8 (cat #101406, BioLegend, San Diego, CA, USA), FITC-anti-mouse CD3 (cat #100203, Biolegend), APC-anti-mouse CD4 (cat #103024, Biolegend), PE-anti-mouse IFNγ^+^ (cat #505808, Biolegend), FITC-anti-mouse CD8 (cat #100705, Biolegend).

### Statistical analysis

Student's t-test, one-way analysis of variance, and Wilcoxon signed-rank test were used. *P* < 0.05 was considered statistically significant.

## Results

### Characterization of sEVs

**Figure 1:**
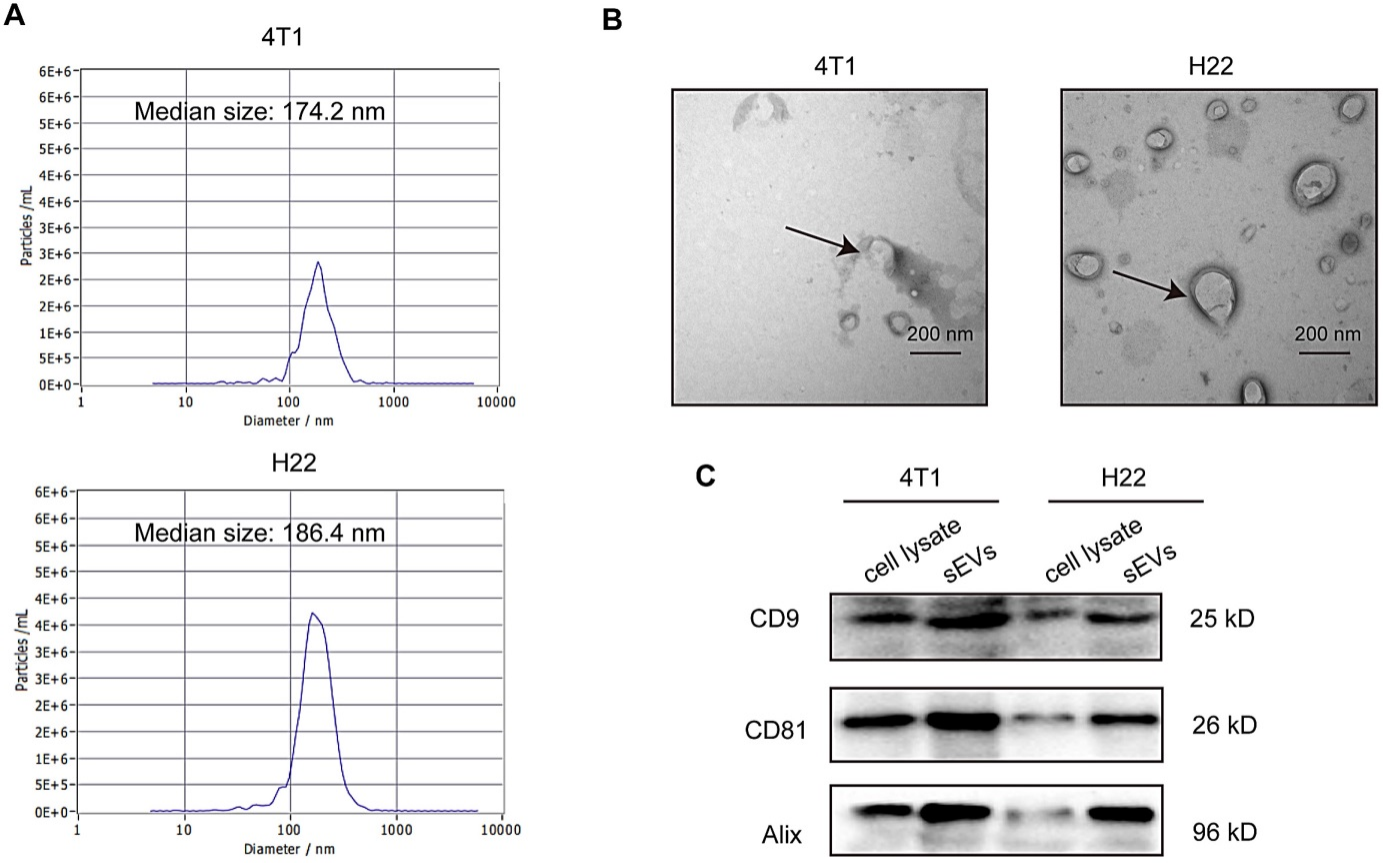
** Characterization of small extracellular vesicles (sEVs). (A)** Particle size distribution measured via nanoparticle tracking analysis (NTA). **(B)** sEV morphology revealed via transmission electron microscopy (TEM).** (C)** Western blot analysis of specific sEV-specific surface markers.

sEVs derived from non-irradiated H22 and 4T1 cells were purified via ultracentrifugation and subjected to TEM, nanoparticle tracking analysis (NTA), and western blotting for characterization. TEM confirmed typical sEVs structures (Figure 1A) and NTA revealed that particle size was mainly distributed around 180 nm (Figure 1B). Furthermore, western blotting revealed the presence of the sEV surface markers CD9, CD81, and Alix (Figure 1C).

### Radiation-induced sEVs triggered antitumor immunity against primary tumor and experimental lung metastasis

**Figure 2:**
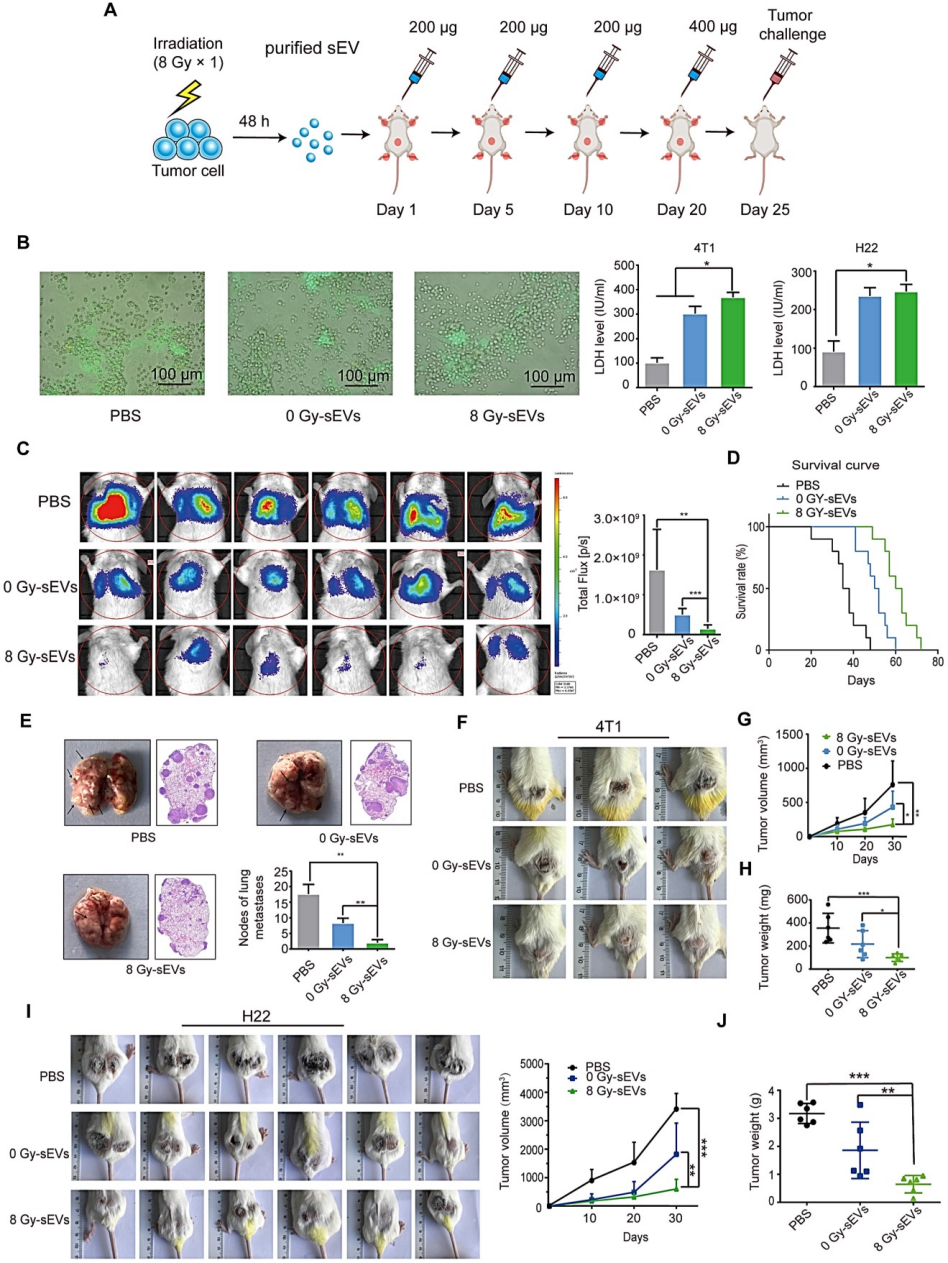
** Antitumor immunity triggered by radiation-induced sEVs against primary tumor and experimental lung metastasis. (A)** Experimental schema depicting sEV purification and immunization. H22 cells and 4T1 cells were subjected to 8 Gy irradiation, and sEVs derived from 8 Gy-irradiated cells (8 Gy-sEVs) or non-irradiated cells (0 Gy-sEVs) were purified via ultracentrifugation. ICR and BALB/c mice were immunized with 200 μg of each type of sEV four times at 5-day intervals and challenged by injection of tumor cells. **(B)** Cell-cell contact between green fluorescent protein (GFP)-positive 4T1 cells and lymphocytes was imaged under fluorescent reverse-microscopy. In a co-culture of 4T1 tumor cells and lymphocytes, 4T1 tumor cells were closely surrounded by lymphocytes from mice immunized with 8 Gy-sEVs, although this was not observed in lymphocytes from mice immunized with 0 Gy-sEVs or the control group. The killing effect was measured as the amount of lactate dehydrogenase (LDH) released (n = 4/group). **(C)** *In vivo* bioluminescence imaging. GFP-luciferase-4T1 cells were intravenously injected into the tail vein of BALB/c mice after immunization. Two weeks later, the growth of 4T1 experimental lung metastases was measured using an IVIS Lumina III *in vivo* imaging system. Luciferase signals from mice immunized with 8 Gy-sEVs were lower than those obtained from the control or 0 Gy group (n = 6/group). **(D)** BALB/c mice were intravenously injected with 4T1 cells via the tail vein. Differences in survival were observed between BALB/c mice immunized with 0 Gy-sEVs, 8 Gy-sEVs, and the control group (n = 10/group). **(E)** Images and hematoxylin & eosin staining of lung metastases and statistical analysis of lung metastatic nodules (n = 6/group). **(F-G)** Primary tumor growth and growth curve of 4T1 breast cancer cells (n = 6/group).** (H)** Tumor weights of 4T1 breast cancer cells at the end of the experiment (n = 6/group). **(I)** Primary tumor growth and growth curve of H22 hepatoma cells (n = 6/group).** (J)** Tumor weights of H22 hepatoma cells at the end of the experiment (n = 6/group). *P*-values were determined using a two-tailed Student's t-test (*P < 0.05, **P < 0.01, ***P < 0.001).

H22 hepatoma and 4T1 breast cancer cells (70% confluence) were subjected to 8 Gy irradiation and the supernatants were collected at 48 h. sEVs derived from 8 Gy-irradiated cells (8 Gy-sEVs) and non-irradiated cells (0 Gy-sEVs) were purified via ultracentrifugation and each type of sEVs (200 μg) was subcutaneously injected into ICR and BALB/c mice four times, at 5-day intervals (Figure 2A). The ICR mice were immunized with sEVs secreted by non-irradiated or 8 Gy-irradiated H22 cells. The BALB/c mice were immunized with sEVs derived from non-irradiated or 8 Gy-irradiated 4T1 cells. Twenty-four days after the first immunization, the immune response elicited against tumors was evidenced by associated cytotoxicity against H22 hepatoma and 4T1 breast cancer cells (Figure 2B).

For the primary tumor model, ICR mice were subcutaneously injected with H22 hepatoma cells and BALB/c mice were subcutaneously injected with 4T1 breast cancer cells 25 days after priming. For the experimental metastasis model, BALB/c mice were injected with 4T1 cells in the tail vein. Priming with 8 Gy-sEVs caused anti-lung metastatic effects that were confirmed via *in vivo* imaging, showing reduced 4T1 metastatic tumor growth in the lungs of 8 Gy-sEV-immunized mice compared to that in control mice or 0 Gy-sEV-immunized mice, as well as lower photon flux (Figure 2C). Importantly, the active anti-cancer immunity triggered by 8 Gy-sEVs may result in prolonged survival (Figure 2D), thus confirming the promising effectiveness of 8 Gy-sEV-activated immunity. At the end of the experiment, lung tissue harvested from 8 Gy-sEV-immunized mice showed fewer metastases and tumor nodules than that from control mice and 0 Gy-sEV-immunized mice (Figure 2E). 4T1 primary tumor growth was greatly suppressed in 8 Gy-sEV-immunized mice and a slower growth curve as well as smaller tumor size and weight were observed compared to those in control and 0 Gy-sEV-immunized mice (Figure 2F-H).

To investigate whether 8 Gy-sEV-triggered antitumor immunity could act universally against different types of tumors in different models, a H22 hepatoma model was used. Similarly, ICR mice were immunized with PBS, 0 Gy-sEVs, or 8 Gy-sEVs. Similar trends were observed, including the slow growth of primary tumors in the 8 Gy-sEV-immunized group (Figure 2I-J).

### Radiation-induced sEVs enhanced tumor infiltration of CD8 and CD4 lymphocytes

**Figure 3:**
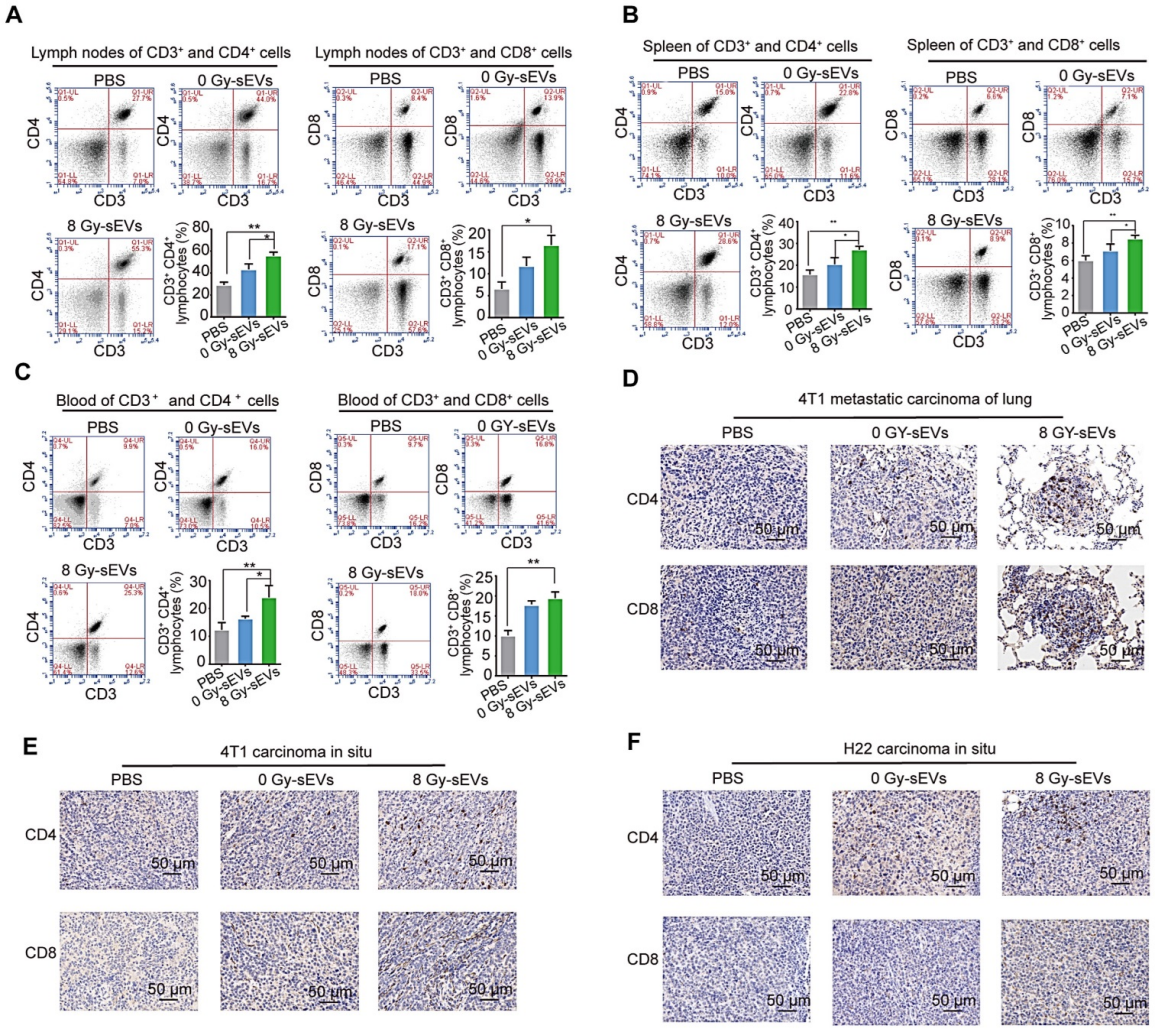
** CD8 and CD4 cell infiltration in 8 Gy-sEV-immunized mice**. **(A-C)** The lymph nodes, spleen, and blood of BALB/c mice were double-stained using anti-mouse fluorescein isothiocyanate (FITC)-anti-mouse CD3, peridinin chlorophyll protein complex (PerCP)-anti-mouse CD8, or allophycocyanin (APC)-anti-mouse CD4, and analyzed using flow cytometry to detect CD4 and CD8. **(D-F)** 4T1 lung metastases, 4T1 primary tumors, and H22 primary tumors were harvested at the end of the experiment and stained with anti-mouse CD8 (imaged at 200× magnification). *P*-values were determined using a two-tailed Student's t-test (*P < 0.05, **P < 0.01, ***P < 0.001).

To explore the cellular mechanisms underlying 8 Gy-sEV-triggered anti-cancer effects, antitumor immune cells (CD4^+^ and CD8^+^ lymphocytes from the lymph nodes, spleen, and blood) were harvested at the end of experiment and analyzed via FCM. The results showed an increase in CD4^+^ and CD8^+^ lymphocytes from the lymph nodes, spleen, and blood in 8 Gy-sEV-treated mice compared to that in control and 0 Gy-sEV-immunized mice (Figure 3A-C). In addition, IHC staining indicated an increase in tumor-infiltrated CD4^+^ and CD8^+^ lymphocytes in both 8 Gy-sEV-immunized ICR and BALB/c mice compared to that in control mice in both models (Figure 3D-F).

### Proteomic and bioinformatic analysis revealed altered cargos in radiation-induced sEVs

Tumor-derived sEVs are carriers that efficiently transfer TAAs to DCs and induce antigen-specific CD4+ and CD8+ T cell activation via reprocessing. Considering that 8 Gy-sEVs induced stronger antitumor immunity than 0 Gy-sEVs, it was hypothesized that radiation may up-regulate a variety of TAAs in sEVs.

**Figure 4:**
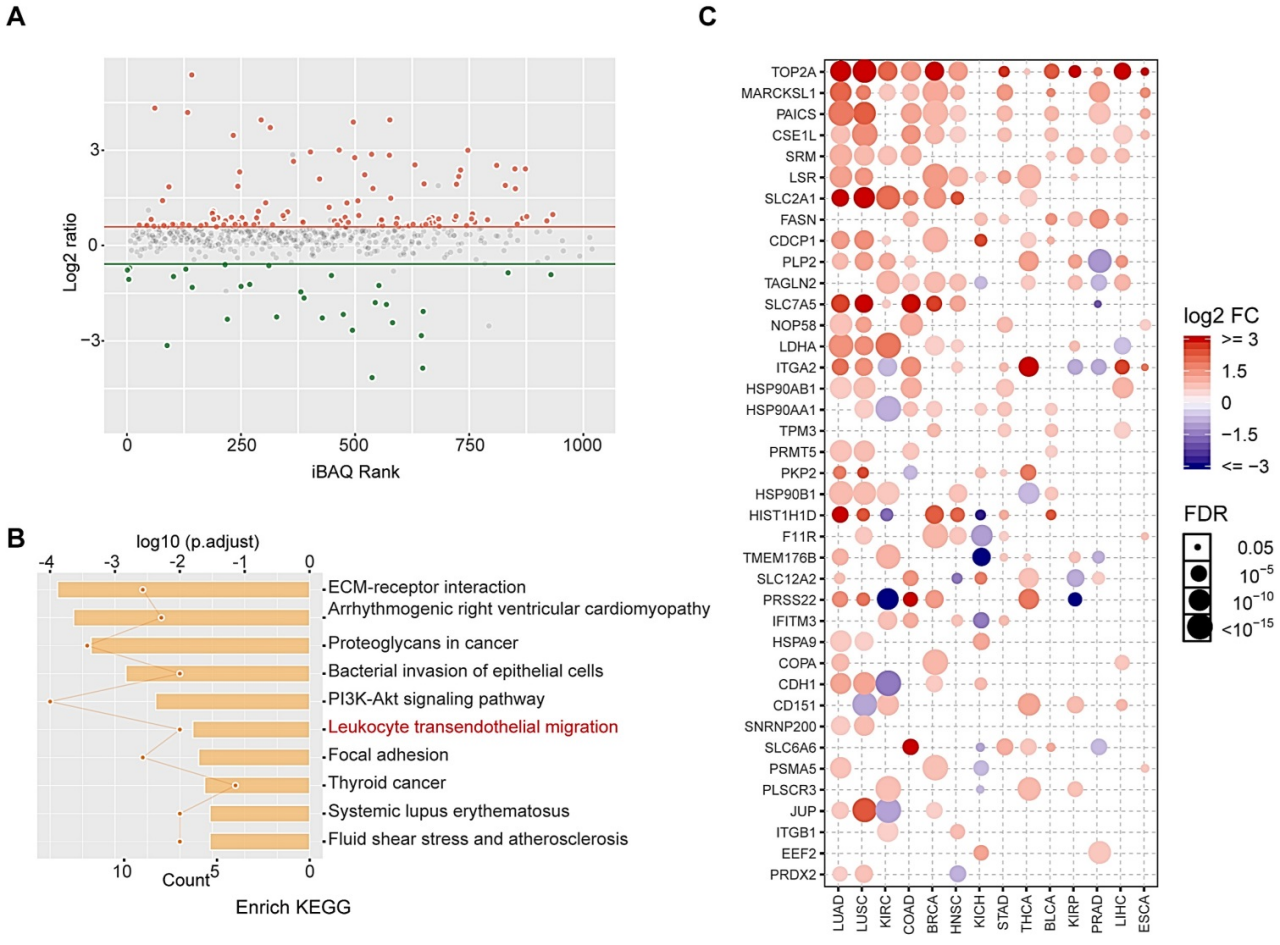
** Proteomic and bioinformatic analysis of altered cargos in radiation-induced sEVs. (A)** Scatter plot showing the distribution of 146 differentially expressed proteins quantified in 8 Gy-sEVs and 0 Gy-sEVs (fold-change > 1.5 and *P* < 0.05). Red dots indicate up-regulated proteins and green dots indicate down-regulated proteins. **(B)** Kyoto Encyclopedia of Genes and Genomes (KEGG) pathway analysis of differentially expressed proteins. **(C)** Expression of up-regulated proteins in various cancers in the Cancer Genome Atlas (TCGA). The intensity of the color represents log2 fold-change (tumor vs normal). Y-axis shows protein name and X-axis shows tumor type, including lung adenocarcinoma (LUAD), lung squamous cell carcinoma (LUSC), kidney renal clear cell carcinoma (KIRC), colon adenocarcinoma (COAD), breast invasive carcinoma (BRCA), head and neck squamous cell carcinoma (HNSC), kidney chromophobe (KICH), stomach adenocarcinoma (STAD), thyroid carcinoma (THCA), bladder urothelial carcinoma (BLCA), kidney renal papillary cell carcinoma (KIRP), prostate adenocarcinoma (PRAD), liver hepatocellular carcinoma (LIHC), and esophageal carcinoma (ESCA).

To identify altered proteins expression in sEVs after radiation, we performed label-free relative quantitative proteomic and bioinformatic analyses. In total, 146 differentially expressed proteins were identified after radiation, including 117 up-regulated proteins and 29 down-regulated proteins (fold-change > 1.5 and *p* < 0.05; Figure 4A). Details regarding altered protein expression are provided in Table S1. Kyoto Encyclopedia of Genes and Genomes (KEGG) pathway analysis revealed that differentially expressed proteins were enriched in leukocyte transendothelial migration signaling pathways (Figure 4B).

Proteins overexpressed in tumor tissues are considered candidate TAAs to prevent serious autoimmune responses. Here, we showed that most up-regulated proteins were overexpressed in various tumors via analysis of the Cancer Genome Atlas (TCGA) database, and these may be regarded as potential TAAs (Figure 4C). These results indicate that radiation may enrich TAAs in sEVs.

We further analyzed the effects of irradiation on the amount of secreted sEVs via nano flow cytometry. Interestingly, the results showed that irradiation promoted the release of sEVs (Figure S1).

### Peptides derived from up-regulated proteins in sEVs triggered antitumor immunity

To be effective molecular targets, the ideal TAAs should show the following three characteristics: tumor over-expression patterns, oncogenicity, and immunogenicity 14**.** To prove that altered proteins in 8 Gy-sEVs may be regarded as potential TAAs, peptides derived from CDCP1 (up-regulated protein in 8 Gy-sEVs ) were designed to prime host antitumor immunity. The rationales for choosing CDCP1 as a tumor associated antigen are as follows:

Firstly, CDCP1, also known as CD318, is a transmembrane protein which is overexpressed on various tumors, including lung, kidney and colon tumors^15^. Here, we revealed that more types of cancers express high levels of CDCP1, including bladder urothelial carcinoma (BLCA), breast invasive carcinoma (BRCA), cervical squamous cell carcinoma and endocervical adenocarcinoma (CESC), colon adenocarcinoma (COAD), glioblastoma multiforme (GBM), kidney chromophobe (KICH), lung adenocarcinoma (LUAD), lung squamous cell carcinoma (LUSC), ovarian serous cystadenocarcinoma (OV), pancreatic adenocarcinoma (PAAD), rectum adenocarcinoma (READ), stomach adenocarcinoma (STAD), testicular germ cell tumors (TGCT), and uterine corpus endometrial carcinoma (UCEC) through a comprehensive analysis of TCGA and Gene Expression Profiling Interactive Analysis (GEPIA) databases (Figure S2A). Secondly, in breast cancer, the expression of CDCP1 mRNA and proteins were differential: high in the tumor tissues and low in adjunct normal tissues (Figure S2B and S2C). Thirdly, CDCP1 expression is associated with cancer stages and progression, which is considered to be barely lost in the process of tumor progression (Figure S2D and S2E). Furthermore, western blot confirmed that CDCP1 expression was up-regulated in 4T1 cells sEVs after radiation (Figure S2F). All data suggest that CDCP1 is likely to be involved in malignant behavior and may be an ideal TAA.

**Figure 5:**
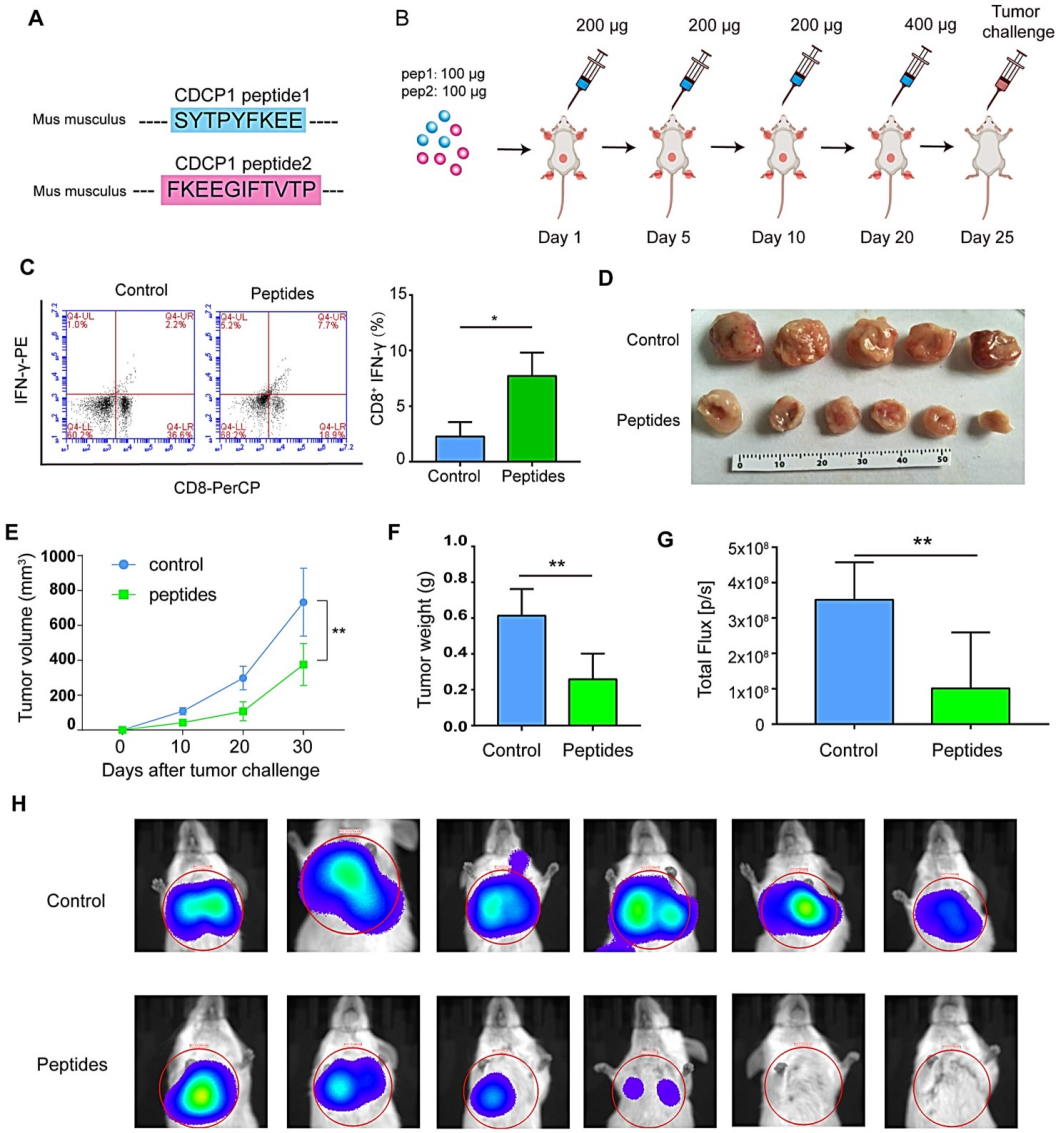
** Peptides derived from up-regulated proteins in sEVs triggered antitumor immunity. (A)** Two peptides derived from CUB domain-containing protein 1 (CDCP1) were synthesized. **(B)** Experiment design. **(C)** Flow cytometry** (**FCM) analysis of differences of CD8^+^-interferon (IFN)γ^+^ T lymphocytes in the control group (phosphate-buffered saline (PBS) with Freund's adjuvant) and peptide group (CDCP1 peptides with Freund's adjuvant; n = 3/group). **(D)** Primary 4T1 tumor sizes in the control group (immunization with PBS four times, at 5-day intervals) and peptide group (immunization with 200 µg CDCP1 peptides four times, at 5-day intervals; n = 6/group). **(E)** Primary 4T1 tumor growth curve in the control (immunization with PBS four times, at 5-day intervals) and peptide-treated group (immunization with 200 µg CDCP1 peptides four times, at 5-day intervals; n = 6/group). **(F)** Primary 4T1 tumor weights (n = 6/group). **(G-H)** Experimental metastasis model and *in vivo* imaging of the lungs in the control group (immunization with PBS four times, at 5-day intervals) and peptide group (immunization with 200 µg CDCP1 peptides four times, at 5-day intervals; n = 6/group). *P*-values were determined using a two-tailed Student's t-test (*P < 0.05, **P < 0.01, ***P < 0.001).

CDCP1 peptides were designed based on optimal MHC I binding and immunogenicity and synthesized (Figure 5A). The experiment progression is depicted in Figure 5B. Twenty-four days after the first immunization, elicited immunity was confirmed by an increase in peptide-stimulated active CD8^+^- IFNγ^+^ T lymphocytes (Figure 5C). Twenty-five days after priming, 1 × 10^6^ 4T1 cells were subcutaneously injected into the backs or caudal veins of BALB/c mice. The growth of 4T1 tumors was greatly suppressed in CDCP1 peptide-immunized mice, and the tumor size was smaller (Figure 5D), the growth curve slower (Figure 5E), the tumor weight lower (Figure 5F), and the photon flux in lung metastasis lower (Figure 5G-H) compared to those in control PBS/Freund's adjuvant-immunized mice.

All data demonstrated that CDCP1 peptides were capable of triggering active immunity to inhibit 4T1 primary and metastatic tumor growth via activation of T lymphocytes. Furthermore, up-regulated CDCP1 in 8 Gy-sEVs may be regarded as a TAA.

### Radiation enriched sEVs with heat shock protein (Hsp)70 and Hsp90

**Figure 6:**
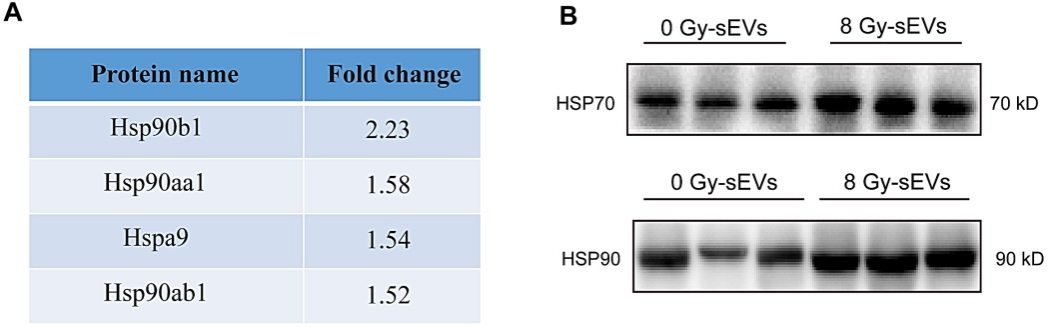
** Heat shock protein (HSP)70 and HSP90 expression in radiation-enriched sEVs. (A)** Proteomic analysis showing up-regulation of the HSP family in sEVs from irradiated 4T1 cells. **(B)** Western blot confirming that Hsp70 and Hsp90 were up-regulated in sEVs from irradiated 4T1 cells.

DAMPs, like stress-induced heat shock proteins (HSPs), act as endogenous “danger signals” that can improve tumor immunogenicity by promoting antigen presentation, activating natural killer cell responses, and inducing cytotoxicity of T-helper cells 16, 17. sEVs represent a novel secretory pathway for HSPs. Here, proteomic results revealed the upregulation of HSPs in 8-Gy radiation-induced sEVs (e.g., Hsp90b1, Hsp90aa1, Hspa9, and Hsp90ab1; Fig. 6A). Furthermore, western blots further validated that Hsp70 and Hsp90 were up-regulated in sEVs by radiation (Fig. 6B). Our results therefore demonstrated that radiation as a powerful stress could up-regulate the expression of Hsp70 and Hsp90 in sEVs, which may improve tumor immunogenicity.

### sEVs immunization enhanced the activation of CTLs via phosphoinositide 3-kinase (PI3K)-protein kinase B (Akt) signaling

**Figure 7:**
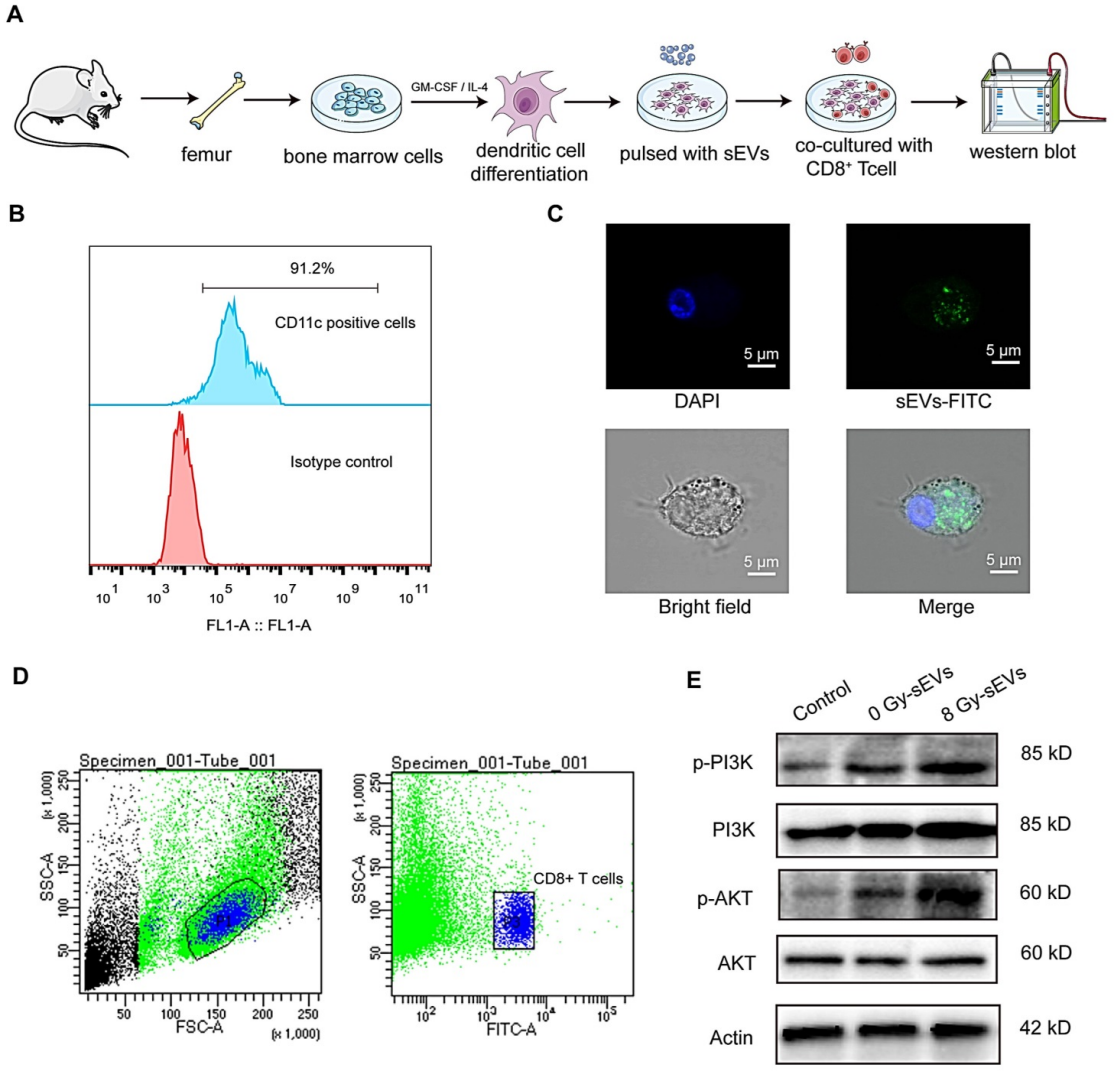
** the underlying mechanism on how sEVs achieve antitumor immunity after radiation. (A)** Experimental design. Bone marrow cells were isolated from the femurs of mice and stimulated with GM-CSF and IL-4 for DCs differentiation. Then, DCs were pulsed with sEVs and co-culture with CD8^+^ T cell. Western bolt was used to analyze activated signaling in CD8+ T cell. **(B)** Detection of CD11c^+^ dendritic cells by FCM after stimulation with granulocyte-macrophage colony-stimulating factor (GM-CSF) and interleukin (IL)-4. **(C)** Uptake of sEVs by DCs. sEVs were first stained with PKH67 dye before 24-h culture with DCs and analysis via confocal microscopy. **(D)** Panels illustrate the gating strategy used for isolation of CD8^+^ T cell subsets via fluorescence-activated cell sorting. **(E)** Western blot revealed the activated signaling in CD8^+^ T cell.

Dendritic cells play a key role in the induction of an efficient and protective adaptive immune response 18. Considering their potent capacity to capture, process, and present antigens to T cells, we investigated the mechanisms underlying 8 Gy-sEVs mediated activation of antitumor immunity starting with the hypothesis that DCs could uptake sEVs and deliver an activation signal to naive T cells. Figure 7A depicted experimental schema. Briefly, DCs derived from bone marrow cells were stimulated with GM-CSF and IL-4. After 7-10 days differentiation, the abundant subsets of CD11c^+^ dendritic cells were detected via flow FCM (Figure 7B). To determine whether DCs could uptake sEVs, sEVs were stained with PKH67 and co-cultured with DCs. As visible by confocal microscopy, the recipient dendritic cells exhibited high efficiency in uptake of the sEVs (Figure 7C).

As PI3K-Akt signaling plays a significant role in the activation of CD8^+^ T cells, we further investigated changes in PI3K-Akt signaling in CD8^+^ cells after co-culture with DCs. Splenic CD8^+^ T cells were isolated via FCM (Figure 7D). Western blotting analysis revealed that DCs primed with 8 Gy-sEVs activated PI3K-Akt signaling in CD8^+^ T cells (Figure 7E). According to these results, 8 Gy-sEVs likely activate CD8^+^ T cells via PI3K-Akt signaling through DC presentation.

## Discussion

**Figure 8:**
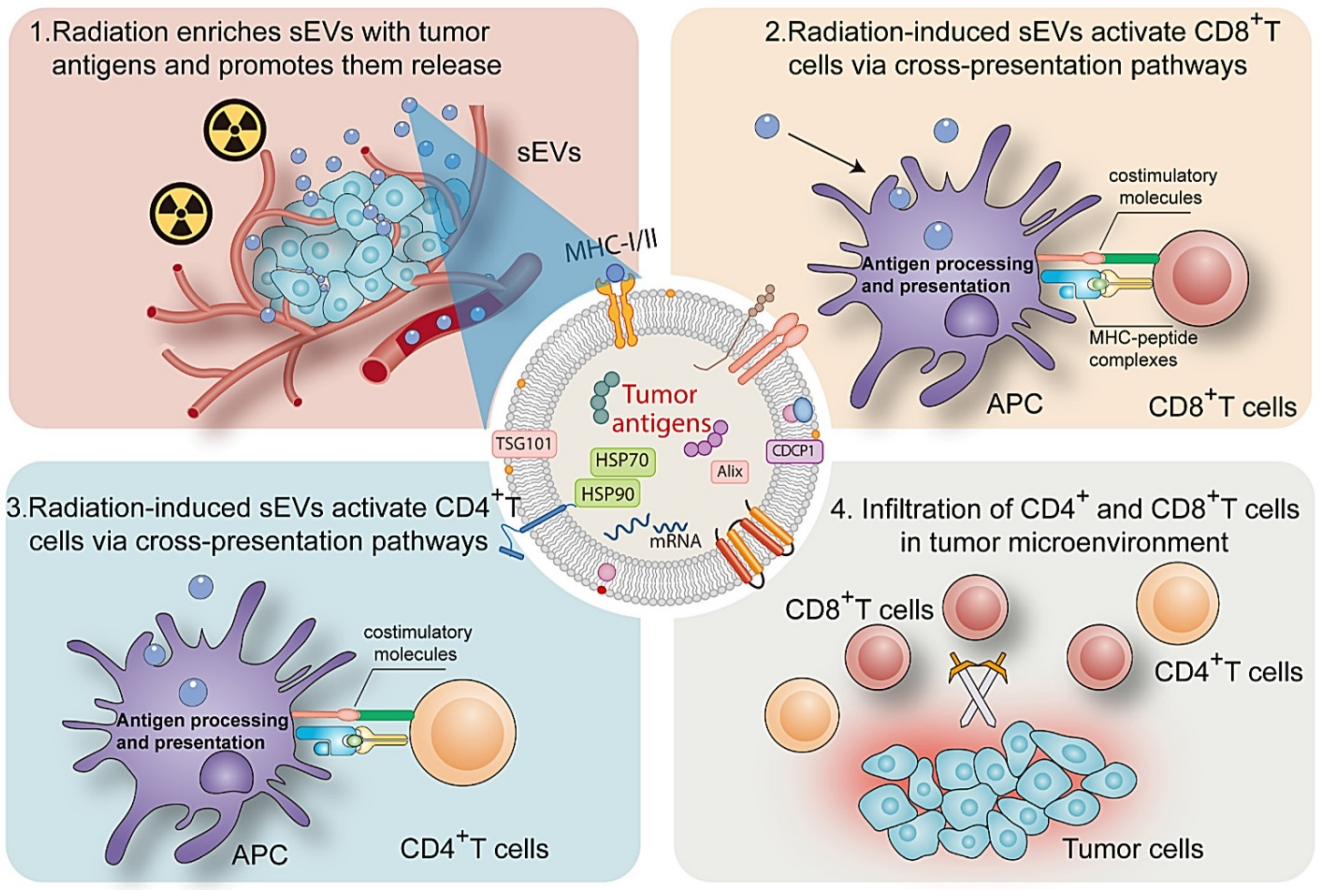
Mechanism by which radiation-induced sEVs trigger antitumor immunity.

sEVs play an important role in cell signaling in numerous cell types. Ranging from 50 to 200 nm in diameter, sEVs secreted by donor cells transport various components (proteins, microRNA, and circular RNA) to recipient cells and regulate cellular physiology 19, 20. A major milestone was achieved in the field of onco-immunology when local tumor irradiation was shown to modulate the immunogenicity of tumor cells 21. Here, as summarized in Figure 8, our results revealed that radiation may enrich sEVs with a variety of TAAs and DAMPs (Hsp70 and Hsp90), and that radiation-induced sEVs may modulate antigen-specific CD4 and CD8 T cell activation via cross-presentation pathways. The induction of antitumor immunity primed by radiation-induced sEVs could represent an effective, long-lasting, and safe approach to suppress cancer progression.

The abscopal effects of RT are mediated by a systemic antitumor immune response and lead to the regression of non-irradiated metastatic lesions at a distance from the primary site of irradiation 2, 21-23. Hypofractionated stereotactic RT (8 Gy) activates antitumor immunity and may have the potential to trigger abscopal effects 24-26. In this study, 8 Gy radiation was used to irradiate tumors and sEVs were harvested for priming. Furthermore, primary tumor and experimental lung metastasis models were established in mice to evaluate the local and metastatic tumor immune microenvironment induced by immunization with sEVs.

Previous studies have reported that sEVs from tumor cells can efficiently deliver TAAs to DCs and have thus been employed as antigen carriers to prime cytotoxic T lymphocytes and elicit an immune response 7. In line with this evidence, our study showed that 0 Gy-sEVs immunization could trigger anti-tumor immunity. Radiation induces distinct tumor cell death processes and, consequently, the release of pro-inflammatory cytokines, TAAs, and DAMPs following cell lysis 27, 28. Radiation may trigger antitumor immunity through this mechanism. Considering the fact that sEVs may be employed as TAAs carriers, we hypothesized that radiation-induced sEVs may activate efficient antitumor immunity by promoting tumor antigen release. Our results revealed that radiation-induced sEVs triggered more efficient antitumor immunity against primary tumor and experimental lung metastases than non-irradiated sEVs. The underlying mechanism may involve the enrichment of tumor antigens and DAMPs such as HSPs in sEVs via radiation.

Down-regulation/loss of antigen presentation is a frequent and important mechanism used by tumor cells to escape immune destruction 29. It confers on tumor cells the capacity to become “invisible” and avoid immune attack, thus limiting the efficacy of cancer immunotherapies. Hence, boosting antigen presentation is an attractive strategy to improve cancer immunotherapy. Functionally, most TAAs promote malignant behaviors and are thus described as oncoproteins 30. Our previous study proved that exposure to radiation up-regulated a panel of oncoproteins, which may be candidate TAAs, in tumor cells and that their immunogenic domains may serve as tumor vaccines to prime host active antitumor immunity 31. Here, we showed that radiation may up-regulate a variety of oncoproteins (overexpressed in tumors) in sEVs, which may increase the immunogenicity of sEVs. Furthermore, peptides derived from sEVs may trigger antitumor immunity, which further validates the abundance of TAAs in sEVs.

Collectively, this study shows that sEVs derived from irradiated tumor cells function as “carriages” that promote TAA release and trigger antitumor immunity. sEVs represent a critical mechanism that contributes to radiation-induced antitumor immunity.

## Supplementary Material

Supplementary figures and table.

## Abbreviations

APC: allophycocyanin
Akt: protein kinase B
BSA: bovine serum albumin
CDCP1: CUB domain-containing protein 1
CTL: cytotoxic T lymphocytes
DAMP: damage-associated molecular pattern
DC: dendritic cell
FACS: fluorescence-activated cell sorting
FBS: fetal bovine serum
FCM: flow cytometry
FITC: fluorescein isothiocyanate
GEPIA: Gene Expression Profiling Interactive Analysis
GFP: green fluorescent proteins
GM-CSF: granulocyte-macrophage colony-stimulating factor
HSP: heat shock protein
IFNγ: interferon-gamma
IHC: immunohistochemistry
IL: interleukin
KEGG: Kyoto Encyclopedia of Genes and Genomes
mAb: monoclonal antibody
MHC: major histocompatibility complex
NTA: nanoparticle tracking analysis
PBS: phosphate-buffered saline
PE: phycoerythrin
PerCP: peridinin chlorophyll protein complex
PI3K: phosphoinositide 3-kinase
RIPA: radioimmunoprecipitation assay
RT: radiation therapy
SDS-PAGE: sodium dodecyl sulfate-polyacrylamide gel electrophoresis
sEVs: small extracellular vesicles
TAA: tumor-associated antigen
TBST: Tris-buffered saline with Tween
TCGA: the Cancer Genome Atlas
TEM: transmission electron microscopy
