# Supplementary material for: Radiation-induced small extracellular vesicles as “carriages” promote tumor antigen release and trigger antitumor immunity
Source: Theranostics. 2020 Mar 26;10(11):4871–84. doi: 10.7150/thno.43539 (PMC7163438; doi:10.7150/thno.43539)
Supplement: Supplementary file 1 — Supplementary figures and table. [file thnov10p4871s1.pdf]

Supplementary materials

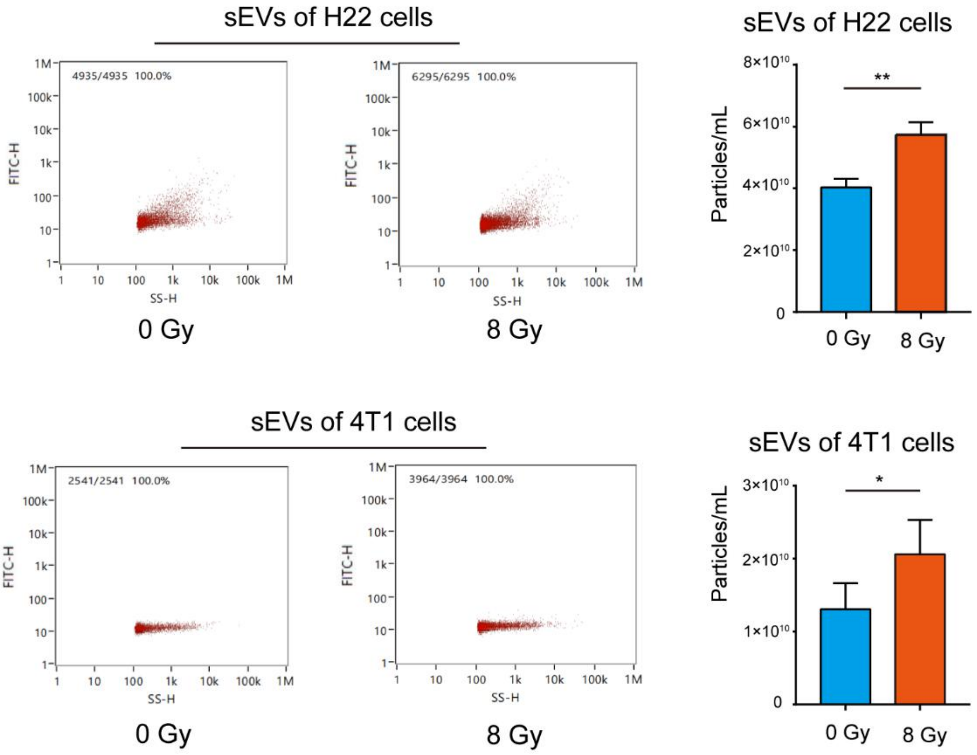

Figure S1. **Effects of irradiation on the amount of secreted small extracellular vesicles (sEVs)**

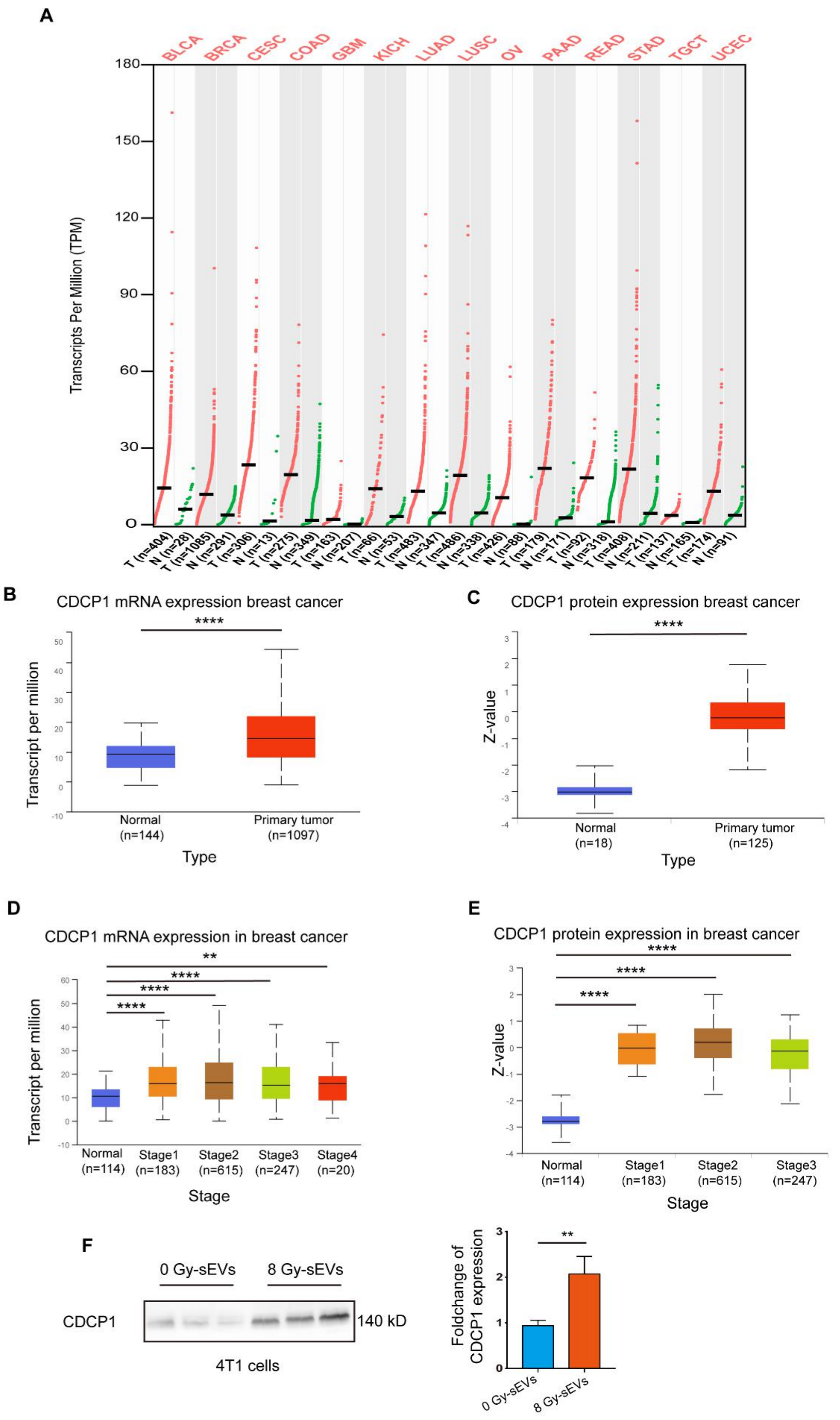

Figure S2. Identification of CDCP1 as an ideal tumor antigen

To identify CDCP1 as an ideal TAA, the Cancer Genome Atlas (TCGA), Gene

Expression Profiling Interactive Analysis (GEPIA), UALCAN, Clinical Proteomic Tumor Analysis Consortium (CPTAC) databases were used for bioinformatic analysis. **(A)** CDCP1 is overexpressed in 14 types of tumors (data from GEPIA and TCGA). **(B)** CDCP1 mRNA expression was over-expressed in breast cancer compared to normal samples (data from UALCAN and TCGA). **(C)** CDCP1 protein expression was over-expressed in breast cancer compared to normal samples (data from UALCAN and CPTAC). **(D-E)** CDCP1 expression was associated with progression stages of breast cancer (data from UALCAN, TCGA and CPTAC). **(F)** CDCP1 expression in 0 Gy-sEVs and 8 Gy-sEVs from 4T1 cells. $^{**}P<0.01$, $^{***}P<0.001$, $^{****}P<0.0001$.

Table S1. Altered proteins in radiation-induced sEVs

	Proteins
IR-upregulated proteins	HIST1H1D, PTTG1IP, CSE1L, VPS35, PRDX2, PRSS22, TPM3-RS7, RALB, SLC7A5, CDCP1, MARCKSL1, PCBP2, MSLN, DNAJA2, NRAS, LSR, COPA, TOP2A, PKP2, QSOX1, PLP2, PSMC3, JUP, RPS11, NOP58, NEDD4L, EPB4.1L2, IFITM3, CPE, PLSCR3, DIP2B, EIF3I, LDHA, SNAP23, GJA1, HSP90B1, DYNAP, AREG, CTSL, GPRC5A, SRM, SNRNP200, PAICS, SLC12A2, PRMT5, CDH1 PRDX6, ARF3, EFTUD2, LAMP1, RPLP0, LAMC2, CPNE8, EIF3B, XPO1, VCL, PSMA4 PGK1-RS7, DNPEP, FARP1, GALK1, RARS, BAIAP2, SEPT2, CTNNB1, PTPRK, GM10259, EZR, NCKAP1, LAMB3, SLC38A2, MYL6, SLC7A1, FAM125A, IPO7, PPID, CD44, PSMB5, LARS, TMEM176B, CTNNA1, WDR1, SLC6A6, SLC2A1, EEF2, PGD, PSMA5, HSP90AA1, F11R, TAGLN2, TSPAN3, SERPINB6A, ITGB1, S100A6, PLET1, FLOT2, FLOT1, SDCBP2, HSPA9, TMPRSS11E, TSPAN4, HNRNPH1, UBA1, SLC44A2, PRPF8, GNAI2, HSP90AB1, EHD1, CD151, ATP2B1, THBS1, ITGA2, FASN
IR-downregulated proteins	C8B, C6, S100A11, ITGA5, H2AFY, WARS, RPL32, RPS12, GNB1, DLAT, HNRNPC, FGG, FABP5, HP, H2AFV, ANP32B, CLU, H2AFJ, SDC4, HINT1, MYBBP1A, GART, HIST4H4, GPC1, HIST2H3B, COL4A2, HTRA1
